# Placental mitochondrial adaptations in preeclampsia associated with progression to term delivery

**DOI:** 10.1038/s41419-018-1190-9

**Published:** 2018-11-19

**Authors:** Olivia J. Holland, James S. M. Cuffe, Marloes Dekker Nitert, Leonie Callaway, Keith A. Kwan Cheung, Filip Radenkovic, Anthony V. Perkins

**Affiliations:** 1 School of Medical Science, Griffith University, Gold Coast Campus, Southport, QLD Australia; 2 School of Biomedical Sciences, The University of Queensland, St Lucia, QLD Australia; 3 Royal Brisbane and Women’s Hospital, University of Queensland Centre for Clinical Research, Herston, QLD Australia; 4 School of Chemistry and Molecular Biosciences, The University of Queensland, St Lucia, QLD Australia; 5 Women and Newborns, Royal Brisbane and Women’s Hospital, Herston, QLD 4101 Australia

## Abstract

Preeclampsia is a devastating pregnancy disorder. Severity varies widely, and while severe preeclampsia often requires pre-term delivery, women with mild preeclampsia may reach term with minor interventions. The mechanisms that mediate disease severity are poorly understood, but may include adaptive processes by the placenta. We aimed to establish whether in pregnancies that reached term and those that delivered pre-term, the placental response to preeclampsia was intrinsically different, and explore potential adaptive mechanisms. Hydrogen peroxide production and antioxidant activity were increased in term preeclamptic placentae, whereas pre-term preeclamptic placentae had reduced hydrogen peroxide production and reduced function of the antioxidant system superoxide dismutase compared to control placentae. Markers of mitochondrial fission/fusion, apoptosis and the expression level of mitochondrial complexes were differentially disrupted in term compared to pre-term preeclamptic placentae. Mitochondrial respiration and content were increased in term preeclamptic placentae, but mitochondria had a lower respiratory reserve capacity. Mitochondrial respiration and hydrogen peroxide production were increased in healthy term placentae after in vitro hypoxia/reoxygenation. Placentae from preeclamptic pregnancies that reached term showed multiple adaptions that were not present in pre-term preeclamptic placentae. Increased antioxidant activity, and expression of markers of mitochondrial fusion and apoptotic suppression, may relate to salvaging damaged mitochondria. Increased mitochondrial respiration may allow ongoing tissue function even with reduced respiratory efficiency in term preeclamptic pregnancies. Response after in vitro hypoxia/reoxygenation suggests that disruption of oxygen supply is key to placental mitochondrial adaptations. Reactive oxygen species signalling in term preeclamptic placentae may be at a level to trigger compensatory antioxidant and mitochondrial responses, allowing tissue level maintenance of function when there is organelle level dysfunction.

## Introduction

Preeclampsia affects 1–5% of pregnancies and is estimated to be responsible for 70,000–80,000 maternal deaths and 500,000 perinatal deaths worldwide every year^1,2^. Preeclampsia has very limited treatment options and can only be cured by delivery. The disorder can therefore require pre-term delivery, which itself is associated with poor offspring health outcomes^2,3^.

Preeclampsia is a multi-system disorder characterised by new onset maternal hypertension and endothelial dysfunction (commonly diagnosed through proteinuria). This broad symptomatic definition likely represents a disorder with complex aetiology. Indeed, sub-types of preeclampsia are recognised in the research literature^4^ and clinical management guidelines^5^. The symptoms of preeclampsia can rapidly become severe enough to result in maternal mortality (e.g., through organ failure and seizures), thus necessitating pre-term delivery as an intervention. Alternatively, the disorder can remain mild enough to allow pregnancy progression to term delivery. The different features of preeclampsia sub-types may represent varied aetiologies or the activation of different damage/repair pathways; however, these differences have only been characterised to a limited degree.

The placenta is central to the pathogenesis of preeclampsia. The precise pathophysiological mechanisms are not fully understood, but there is substantial evidence that early defects in placentation result in perturbed maternal blood flow to the placenta as pregnancy progresses, leading to further placental damage and oxidative stress^6,7^. Disrupted delivery of blood, and therefore oxygen, to a wide range of tissues is known to cause damage to the mitochondrial electron transfer system (ETS), which leads to increased production of reactive oxygen species (ROS)^8^. Antioxidant systems (e.g., superoxide dismutase, catalase, glutathione peroxidase), as well as coordinated mitochondrial (e.g., biogenesis, fission/fusion of mitochondria network) and cellular (e.g., autophagy, apoptosis) adaptations constitute the cellular response to ROS and ROS-induced damage^9,10^. The relative levels of these responses versus ROS levels determine the effectiveness at counteracting damage. Mitochondrial damage and ROS production have been well characterised in the heart following myocardial infarction, and the ability of cardiac tissue to respond to ischaemia then to reperfusion is critical to outcomes^11–13^. Furthermore, the dynamic response of mitochondria can lead to adaptive changes in tissues to better suit cellular metabolism to the environment. For example, in skeletal muscle, exercise enhances mitochondrial respiration and biosynthesis^14,15^, and in cardiac muscle, exposure to low levels of ROS before a major insult results in a range of adaptions that lead to better outcomes (termed ischaemic preconditioning)^16–18^.

There is evidence that severe preeclampsia is associated with increased ROS and reduced placental antioxidant function^19,20^, and in vitro antioxidant treatment can improve trophoblast responses to oxidative stress^21–23^. However, clinical trials of antioxidants for the treatment of preeclampsia have been generally unsuccessful^24,25^, and ROS-mediated signalling may be important for maintaining placental function^26^.

To investigate if the less severe forms of preeclampsia represent successful countering of adverse conditions, the current study compares antioxidant function and mitochondrial dynamics between placentae from control and preeclamptic pregnancies that either reached term or were delivered pre-term. The direct effect of hypoxia/reoxygenation on placental mitochondria is also investigated in healthy placental tissue.

## Materials and methods

### Materials, tissue collection, and ethics

Chemicals were purchased from Sigma Aldrich, Australia unless noted otherwise. This study was approved by the Queensland Government Human Research Ethics Committee, Australia (HREC/08/QRBW/1 and HREC/14/QPCH/246), Griffith University (MSC/05/15/HREC), and the University of Queensland (UQ 2009000115). All tissues were obtained following written informed consent. Preeclamptic pregnancies (term delivery *n* = 14, pre-term delivery *n* = 8) were diagnosed following American College of Obstetrics and Gynecology criteria. Control pregnancies (term delivery *n* = 20, pre-term delivery *n* = 10) were matched for gestational age, maternal age, maternal BMI, and baby weight. Pre-term delivery was defined as < 37 completed weeks of gestation. Exclusion criteria for controls was hypertension (pre-existing or onset during pregnancy) and/or proteinuria, as defined following American College of Obstetrics and Gynecology criteria. Participants’ details are given in Table 1. Placentae were collected from a separate cohort of healthy term pregnancies (*n* = 11) for in vitro experiments. Tissues were collected as previously described^27^.

**Table 1:** Patient parameters

	Term			Pre-term		
	Control	Preeclamptic		Control^a^	Preeclamptic	
Gestational age (weeks)	38.75 ± 0.84	37.88 ± 2.10	NS	29.29 ± 3.83	29.73 ± 3.21	NS
Maternal age (years)	27.47 ± 3.93	29.70 ± 7.73	NS	28.23 ± 6.29	32.64 ± 6.28	NS
Maternal BMI (kg/m^2^)	25.17 ± 3.84	27.39 ± 5.83	NS	28.88 ± 7.72	28.99 ± 11.01	NS
Baby weight (kg)	3.26 ± 0.36	3.23 ± 0.80	NS	1.37 ± 0.65	1.25 ± 0.63	NS
SBP (mmHg)	120.78 ± 6.5	149.30 ± 7.5	****	118.6 ± 17.0	167.36 ± 17.6	****
DBP (mmHg)	74.44 ± 6.2	88.00 ± 11.1	**	71.5 ± 14.7	103.6 ± 13.3	****
IUGR (%)	0	9	–	50	38	–
Labour (%)	100%	100%	–	36%	0%	–
Mode of delivery	100% VD	38% VD	–	36% VD	0% VD	–
	0% CS	62% CS^b^	–	63% CS	100% CS	–
Sex of baby (male/female), %	50/50	44/55	–	50/50	63/37	–

*Note*: Data presented as mean ± standard deviation

*BMI*body mass index, *SBP*systolic blood pressure, *DBP*diastolic blood pressure, *VD*vaginal delivery, *CS*caesarean section, *NS*non-significant

^a^Reasons for pre-term delivery in the pre-term control group included pre-term premature rupture of the membranes, intrauterine growth restriction, hind water leak, hyperemesis, cholestasis, infection, and Factor V Leiden mutation

^b^All term preeclamptic caesarean section deliveries were laboured

**P < 0.01, ****P < 0.0001

### Gene expression

RNA was extracted using the RNeasy mini kit (Qiagen, Australia) and reverse transcribed using the iScript gDNA clear cDNA synthesis kit (Biorad, Australia), following manufactures’ protocols. qPCR was performed using the Quanti-Nova SYBR Green PCR Kit (Qiagen) following manufacture’s protocol. Expression of *SOD1*, *SOD2*, *DMN1L*, *OPA1*, *BACE1*, *BID*, *PSENEN*, *CASP3*, *CASP8*, *ADAM10*, *XIAP,* and *BCAP31* were analysed using Kiqstart SYBR Green primers (Sigma Life Science; primer pair one). Gene expression was normalised to the geometric mean of two loading controls (beta actin and beta-2 microglobulin; these genes were stable across all groups, within two standard deviations of the mean) and expression calculated using the 2^−ΔΔCt^ method.

### Total protein extraction, mitochondrial fraction isolation, and protein estimation

Approximately 30 mg of tissue was homogenised in radioimmunoprecipitation buffer (100 mM Tris, 300 mM NaCl, 10% NP-40, 10% sodium deoxycholate, 1% sodium dodecyl sulphate, protease inhibitor, 200 mM phenylmethanesulfonyl fluoride) in a glass homogeniser then centrifuged at 13,200×*g* for 5 min and supernatant collected.

Crude mitochondrial enriched fractions were isolated based on published methodology^28–30^. Briefly, approximately 30 mg of tissue was homogenised in isolation media (250 mM sucrose, 0.5 mM Na_2_EDTA, 10 mM Tris, pH 7.4) then centrifuged at 1500×*g* for 10 min. The supernatant was centrifuged at 4000×*g* for 15 min to separate the mitochondrial pellet.

Protein content was determined using the Pierce BCA Protein Assay Kit (Thermo Fisher Scientific, Australia) following manufacture’s protocol.

### Western blotting

Protein was standardised to a concentration of 1 µg/µL in loading buffer (62.5 mM Tris pH 6.8, 2% sodium dodecyl sulphate, 10% glycerol, 5% β-mercaptoethanol, 0.001% bromophenol blue) and denatured by heating. 20 µg of protein was loaded into 12% polyacrylamide gels for separation by electrophoresis, and proteins transferred onto polyvinylidene fluoride membranes (Merck, Australia). Membranes were blocked with Odyssey Blocking Buffer (Millennium Science, Australia) and incubated overnight with primary antibodies (Table 2). Membranes were incubated with secondary antibodies (1:10,000; IRDye 680 or IRDye 800CW; LI-COR, Australia) for one hour and protein expression visualised using the Odyssey CLX (LI-COR). The level of the protein of interest was normalised to consistent protein loading control (beta actin, glyceraldehyde 3-phosphate dehydrogenase, or complex V).

**Table 2:** Primary antibodies used in western blotting

Protein target	Manufacturer	Catalogue number	Concentration used
DRP1	Abcam	ab56788	1:500–1:1,000
FIS1	GeneTex	GT4211	1:1,000
OPA1	Abcam	ab119685	1:1,000
MFN1	Abcam	ab57602	1:1000
MFN2	Abcam	ab56889	1:200–1:500
BAX	Abcam	ab32503	1:500
BCL2	Abcam	ab32124	1:500
CASP9	Bioss Antibodies	bs-0049R	1:1,000
Cleaved CASP3	Cell Signalling	Asp175	1:1,000
NFκB	Cell Signalling	D14E12	1:1,000
ETS complexes	Abcam	ab110411	1:500–1:1,000
β-actin	Abcam	ab8226	1:1,000
GAPDH	Abcam	Ab9485	1:2,500

### Hydrogen peroxide production

Amplex Ultra Red (Thermo Fisher Scientific) was used to detect hydrogen peroxide (H_2_O_2_) levels in protein lysates following the manufacture’s protocol.

### Antioxidant activity

Total antioxidant activity in protein lysates was measured following published methodology^31,32^. Briefly, protein lysates standardised to a concentration of 900 µg/mL or trolox standards were added to oxidised 2,2’-Azino-di-[3-ethylbenzthiazoline sulphonate] and reduction spectrophotometrically measured. Results are expressed relative to trolox activity (mmol/L trolox equivalent). Superoxide dismutase activity was determined in protein lysates using the Superoxide Dismutase Assay Kit (Cayman Chemical, Australia) following the manufacturer’s protocol. Catalase activity was determined in protein lysates using the Catalase Activity Assay Kit (Abcam, Australia) following the manufacture’s protocol. Glutathione peroxidase (GPx) activity was determined in protein lysates following published methodology^33^.

### Citrate synthase activity

Citrate synthase activity was determined in protein lysates following published methodology^34^.

### MtDNA/nDNA ratio

The mtDNA/nDNA ratio was measured as previously published^27^. Briefly, qPCR was performed using mtDNA or nDNA primer sets (Table 3), and content calculated as the ratio of mtDNA to nDNA using the 2^−ΔΔCt^ method.

**Table 3:** Primer sequences for mitochondrial content determination

Target	Name	Primer sequence	Reference
nDNA	MTAIB_f	GAG TTT CCT GGA CAA ATG AG	^69^
	MTAIB_r	CAT TGT TTC ATA TCT CTG GCG	
nDNA	MTBA_f	AGC GGG AAA TCG TGC GTG AC	^69^
	MTBA_r	AGG CAG CTC GTA GCT CTT CTC	
mtDNA	MTRT4_f	ATG GCC CAC CAT AAT TAC CC	^69^
	MTRT4_r	CAT TTT GGT TCT CAG GGT TTG	
mtDNA	MTRT5_f	GCC TTC CCC CGT AAA TGA TA	^70^
	MTRT5_r	TTA TGC GAT TAC CGG GCT CT	

### High-resolution respirometry

Mitochondrial respiration was assessed as previously described^27^. Briefly, the plasma membrane of tissue samples was permeabilized with 50 μg/mL saponin in 1 mL BIOPS solution (2.77 mM CaK_2_EGTA, 7.23 mM K_2_EGTA, 5.77 mM Na_2_ATP, 6.56 mM MgCl_2_⋅6H_2_O, 20 mM taurine, 15 mM Na_2_ phosphocreatine, 20 mM imidazole, 0.5 mM dithiothreitol, 50 mM MES, pH 7.1) for 30 min on ice, washed twice for 10 min on ice in MiR05 respiration medium (0.5 mM EGTA, 3 mM MgCl_2_·6H_2_O, 60 mM K-lactobionate, 20 mM taurine, 10 mM KH_2_PO_4_, 20 mM HEPES, 110 mM sucrose, 1 g/L BSA essentially fatty acid free, pH 7.1) and blot-dried before measuring wet weight. To measure mitochondrial respiration, 10—20 mg wet weight of tissue was added to an Oxygraph-2k respirometer (Oroboros Instruments, Austria) chamber containing MiR05 at 37 °C. The activity of specific respiratory states was determined by the addition of ETS substrates and inhibitors; pyruvate (5 mM), glutamate (10 mM), and malate (2 mM) were added to determine complex I (CI) mediated LEAK respiration, then oxidative phosphorylation (OXPHOS) through CI was stimulated by the addition of ADP (1–5 mM). Cytochrome c (10 μM) was then added to test the integrity of the outer mitochondrial membrane. Succinate (10 mM) addition then stimulated OXPHOS through complex II (CII), and this was followed by titration of the uncoupler carbonyl cyanide m-chloro phenyl hydrazone (CCCP; 1 mM) to investigate ETS capacity with CII mediated flux alone. Rotenone (1 μM) was added to inhibit CI. The addition of antimycin A inhibited complex III (CIII), and residual oxygen consumption was measured (indicating non-ETS respiration). N,N,N′,N′-tetramethyl-p-phenylenediamine dihydrochloride (TMPD; 0.5 mM) and ascorbate (2 mM) were added to determine OXPHOS through complex IV.

### In vitro hypoxia/reoxygenation with measurement of mitochondrial respiration and hydrogen peroxide production

Samples from the same placenta were assessed simultaneously, with one sample maintained at atmospheric oxygen while the other was allowed to respire in anoxia. Anoxia was maintained for 20 min before reintroduction of oxygen. Amplex Ultra Red (Thermo Fisher Scientific) was used to detect H_2_O_2_ levels in placental tissue during respiration following published methodology^35^.

### Statistical analysis

All values are mean ± SD. PCR and western blot data is presented normalised to healthy term controls. For activity assays, PCRs and western blots, effect of preeclampsia, gestational age and the interaction of these factors was determined with two-way ANOVA with Tukey’s multiple comparisons test. For western blots, representative samples from term and pre-term groups were included in each experiment within gestational groups, but not between gestational groups. For respirometry, normality was met and the effect of preeclampsia was determined with *t*-test. Effect of hypoxia/reperfusion was determined with paired *t*-test, or Wilcoxon test if normality was not met. Significance level was set at 0.05. Analyses were performed using GraphPad PRISM 7.02 (GraphPad, USA).

## Results

### Hydrogen peroxide production and signalling response

**Fig. 1:**
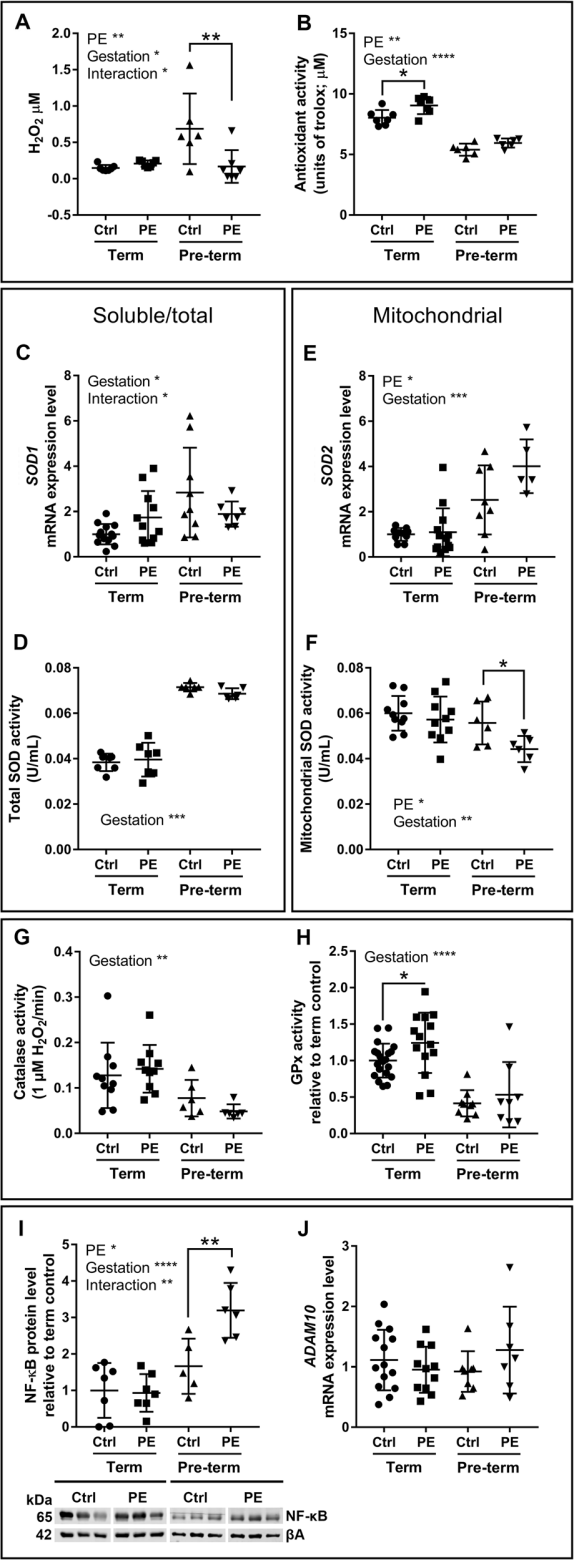
Total antioxidant activity increases in term preeclamptic placentae and superoxide dismutase function is reduced in pre-term preeclamptic placentae. **a** Hydrogen peroxide (H_2_O_2_) level was affected by preeclampsia (*p* = 0.0295), gestation (*p* = 0.0186) and interaction factor (*p* = 0.0074), with levels decreased in pre-term preeclamptic placentae compared to pre-term controls (*p* = 0.0014, fold change 0.244). **b** Total antioxidant activity was affected by preeclampsia (*p* = 0.0024) and gestation (*p* < 0.0001), with activity increased in term preeclamptic placentae compared to term controls (*p* = 0.0171, fold change 1.130). **c** Soluble superoxide dismutase 1 (*SOD1*) mRNA expression was affected by gestation (*p* = 0.0130), and the interaction of preeclampsia/gestation (*p* = 0.0329). **d** Total superoxide dismutase (SOD) activity over all cellular compartments was affected by gestation (*p* < 0.0001). **e** Mitochondrial superoxide dismutase 2 (*SOD2*) mRNA expression was affected by preeclampsia (*p* = 0.0310) and gestation (*p* = 0.0001). **f** SOD activity in enriched mitochondrial fractions was affected by preeclampsia (*p* = 0.0305) and gestation (*p* = 0.0100), and was reduced in pre-term preeclamptic placentae compared to pre-term controls (*p* = 0.0277, fold change 0.794). **g** Catalase activity was affected by gestation (*p* = 0.0010). **h** Glutathione peroxidase (GPx) activity was affected by gestation (*p* < 0.0001) and increased in term preeclamptic placentae compared to term controls (*p* = 0.0357, fold change 1.244). **i** Nuclear factor kappa-light-chain-enhancer of activated B cells (NF-κB) was affected by preeclampsia (*p* = 0.0164), gestation (*p* < 0.0001), and interaction factor (*p* = 0.0095), and was increased in pre-term preeclamptic placentae (*p* = 0.0077, fold change 1.599). **j** mRNA expression of ADAM metallopeptidase domain 10 (*ADAM10*) was not different between groups. βAbeta actin. Representative western blot images. *N* = 5–20; *P < 0.05; **P < 0.01; ***P < 0.001

To investigate ROS, antioxidants, and signalling responses in placentae from preeclamptic pregnancies that reached term versus those that delivered pre-term, H_2_O_2_ production, antioxidant function, and ROS-related signalling were assessed in matched placentae from control and preeclamptic pregnancies (Table 1; Fig. 1). Preeclampsia and gestational age affected both H_2_O_2_ production and total antioxidant activity. In placentae from term preeclamptic pregnancies, there was an increase in total antioxidant activity compared to controls, whereas a decrease in H_2_O_2_ production and no change in total antioxidant activity were observed in pre-term preeclamptic placentae compared to controls (Fig. 1a, b).

Preeclampsia and gestational age affected markers of superoxide dismutase (SOD) function (Fig. 1c–f). There were no changes in markers of SOD function between healthy term and preeclamptic term placentae. In pre-term preeclamptic placentae, the expression of the mitochondrial-targeted *SOD2* was not changed; however, SOD activity in enriched mitochondrial fractions was reduced in pre-term preeclamptic placentae compared to controls (Fig. 1e, f). Catalase activity (fold change 0.467; Fig. 1g) and glutathione peroxidase (GPx; fold change 0.422; Fig. 1h) activity were decreased in pre-term compared to term placentae. GPx activity was increased in placentae from term preeclamptic pregnancies compared to controls.

Levels of the immune and apoptotic regulator nuclear factor kappa-light-chain-enhancer of activated B cells (NF-κB) were unchanged in term preeclamptic placentae and increased in pre-term preeclamptic placentae compared to controls (Fig. 1i). The α-secretase ADAM metallopeptidase domain 10 (*ADAM10*; Fig. 1j), β-secretase 1 (*BACE1*) and presenilin enhancer, gamma-secretase subunit (*PSENEN*) were not different between groups (Supplementary Fig. 1).

### Mitochondrial fission/fusion

**Fig. 2:**
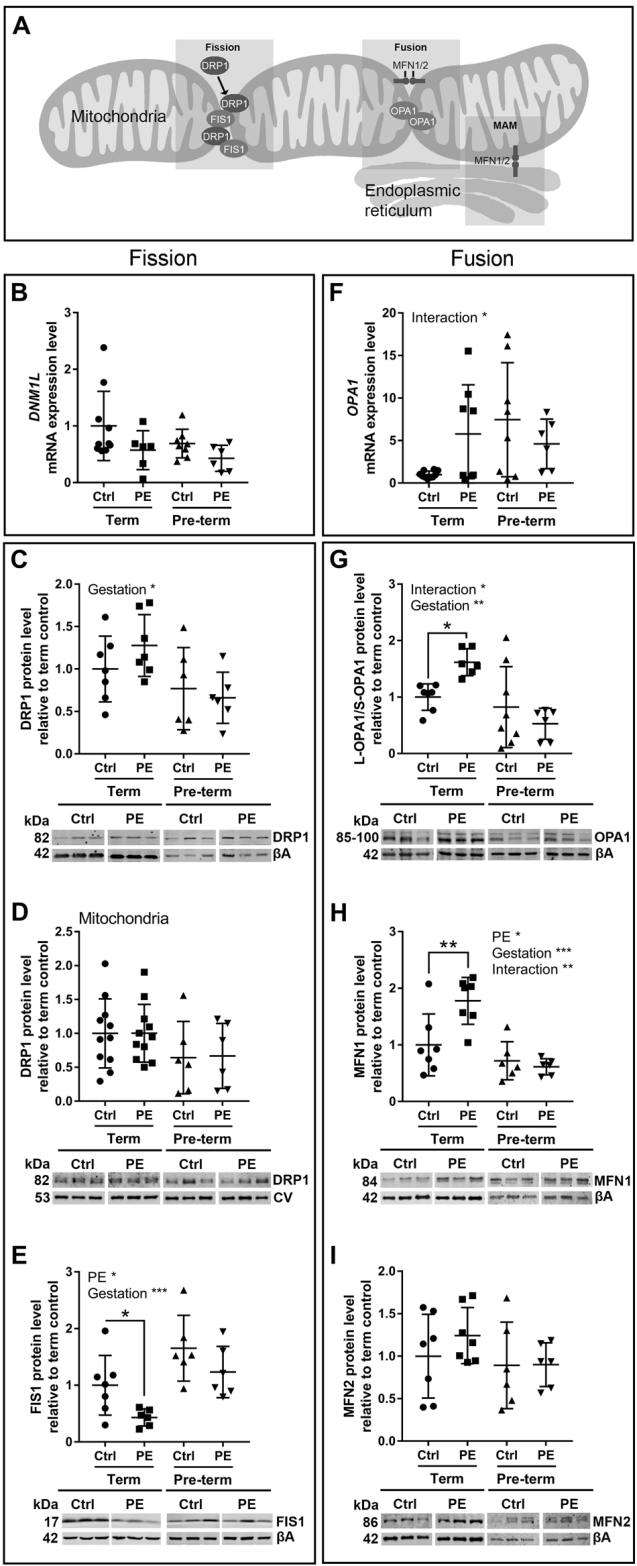
Mitochondrial fission/fusion signalling is disrupted differentially in term and pre-term preeclamptic placenta. **a** Diagram of proteins involved in mitochondrial fusion/fission processes. **b** mRNA expression, (**c**) total protein and (**d**) mitochondrial protein fractions of pro-fission dynamin-1-like protein (mRNA = *DNM1L*; protein = DRP1) were unchanged between control and preeclamptic placentae, with DRP1 affected by gestation (*p* = 0.0122). **e** Mitochondrial fission 1 protein (FIS1) was affected by preeclampsia (*p* = 0.0145) and gestation (*p* = 0.0008), and was decreased in term preeclamptic placentae (*p* = 0.0384, fold change 0.406). **f** mRNA expression of pro-fusion mitochondrial dynamin like GTPase (*OPA1* /OPA1) was affected by the interaction of preeclampsia and gestation (*p* = 0.0288). **g** The ratio of the OPA1 long isoform to short isoform (L-OPA1/S-OPA1) was affected by gestation (*p* = 0.0013) and interaction factor (*p* = 0.0147), and was increased in term preeclamptic placentae (*p* = 0.0207, fold change 1.617) and unchanged in pre-term preeclamptic placentae. **h** Mitofusin 1 (MFN1) was affected by preeclampsia (*p* = 0.0436) and gestation (*p* = 0.0001) and interaction factor (*p* = 0.0099), and was increased in term preeclamptic placentae (*p* = 0.007, fold change 1.778), (**i**) with no difference in mitofusin 2 (MFN2). MAMmitochondria associated membrane; βAbeta actin; CVmitochondrial complex V. Representative western blot images. *N* = 6–7; *P < 0.05; **P < 0.01; ***P < 0.001

To investigate if the response of placental mitochondria to preeclampsia was different between term and pre-term pregnancies, modulators of mitochondrial fission/fusion processes were investigated (Fig. 2a). There were no preeclampsia-associated changes in pro-fission dynamin-1-like protein (*DMN1L*/DRP1) at the gene (Fig. 2b) or protein (Fig. 2c and d) level in any groups. In term preeclamptic placentae, there was a decrease in fission 1 protein (FIS1) compared to controls (Fig. 2e). Pro-fusion mitochondrial dynamin like GTPase (*OPA1*) expression was affected by gestational age and interaction of preeclampsia/gestational age (Fig. 2f). There was no change in OPA1 total protein levels (Supplementary Fig. 2), but a relative increase in OPA1 long isoform (L-OPA1) compared to the short isoform (S-OPA1; Fig. 2g), and pro-fusion mitofusin 1 (MFN1) levels were increased (Fig. 2h), with no change in mitofusin 2 (MFN2), in term preeclamptic placentae compared to controls (Fig. 2i).

### Mitochondrial-linked apoptotic signalling

**Fig. 3:**
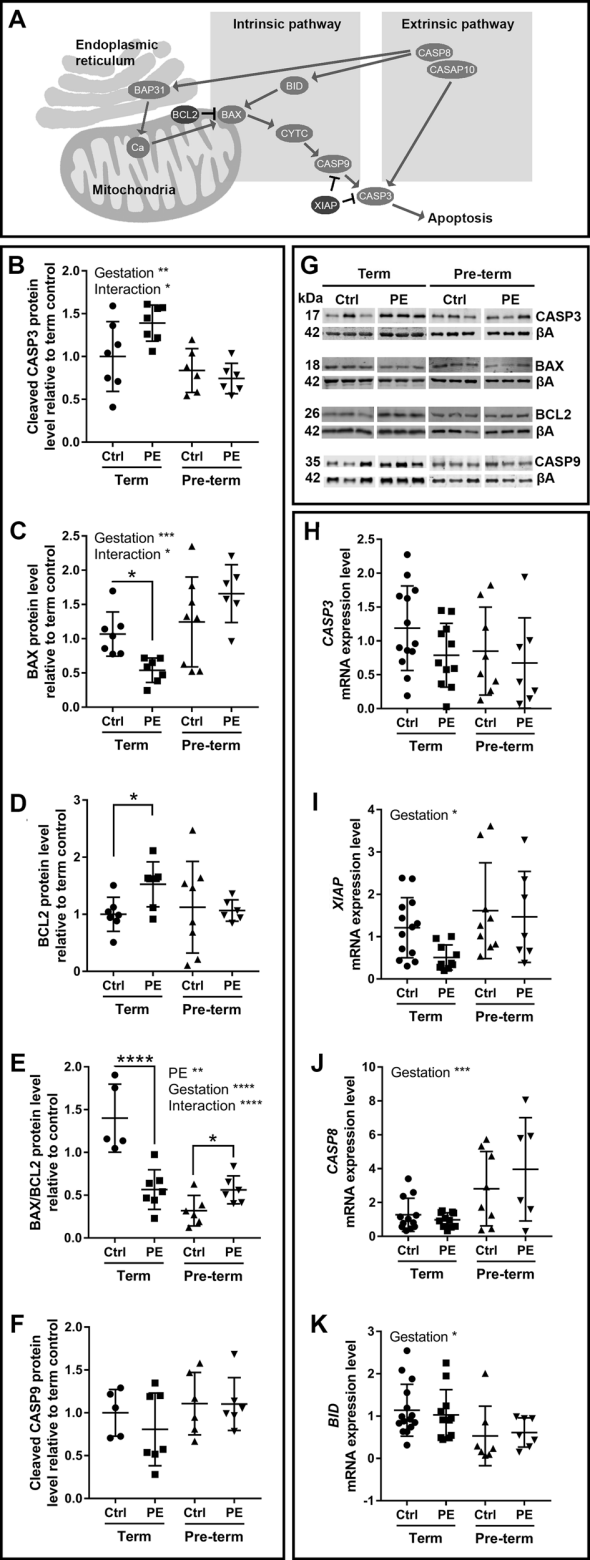
Mitochondrial apoptotic signalling is disrupted differentially in term and pre-term preeclamptic placenta. **a** Diagram of proteins involved in mitochondrial apoptotic signalling. **b** Pro-apoptotic cleaved caspase 3 (CASP3) was affected by gestation (*p* = 0.0014) and interaction factor (*p* = 0.0403). **c** Pro-apoptotic Bcl-2-associated X protein (BAX) was affected gestation (p = 0.0008) and interaction factor (*p* = 0.0101), and was reduced in term preeclamptic placentae (*p* = 0.0356, fold change 0.5394). **d** Anti-apoptotic B-cell lymphoma 2 (BCL2) was increased in term preeclamptic placentae (*p* = 0.0156, fold change 1.526). **e** The BAX/BCL2 ratio was affected by preeclampsia (*p* = 0.0093), gestation (*p* < 0.0001) and interaction factor (*p* < 0.0001), and was decreased in term preeclamptic placentae (*p* < 0.0001, fold change 0.4044) and increased in pre-term preeclamptic placentae (*p* = 0.0334, fold change 1.763). **f** Pro-apoptotic cleaved caspase 9 (CASP9) did not change. **g** Representative western blot images. **h** mRNA expression of *CASP3* was not different between groups. mRNA expression of (**i**) X-linked inhibitor of apoptosis protein (*XIAP*), (**j**) caspase 8 (*CASP8*), and (**k**) BH3 interacting-domain death agonist (*BID*) were affected by gestation (*p* = 0.0172, 0.0004, 0.0136, respectively). BAP31B-cell-receptor-associated protein 31; Cacalcium; CASP10caspase 10; CYTCcytochrome c; βAbeta actin. *N* = 6–8; *P < 0.05; **P < 0.01; ***P < 0.001

As mitochondrial processes are closely linked to the coordination of apoptosis (Fig. 3a), apoptotic related signalling molecules were investigated (Fig. 3). There were no changes in levels of the execution-phase apoptotic protein cleaved caspase 3 (CASP3; Fig. 3b), and also no changes in *CASP3* mRNA expression (Fig. 3h) between preeclamptic placentae and controls. CASP3 was affected by gestational age, and the interaction of preeclampsia/gestational age (Fig. 3b). Pro-apoptotic Bcl-2-associated X protein (BAX; Fig. 3c) was reduced and anti-apoptotic B-cell lymphoma 2 (BCL2; Fig. 3d) was increased in term preeclamptic placentae compared to term controls. The BAX/BCL2 ratio was reduced in in term preeclamptic placentae compared to term controls, and increased in pre-term preeclamptic placentae compared to pre-term controls (Fig. 3e). There were no changes in the mitochondrial-linked apoptotic protein cleaved caspase 9 (CASP9; Fig. 3f) or expression of the endoplasmic reticulum (ER) protein B-cell-receptor-associated protein 31 (*BAP31*; Supplementary Fig. 3). Expression of the anti-apoptotic X-linked inhibitor of apoptosis protein (*XIAP*), and pro-apoptotic caspase 8 (*CASP8*) and BH3 interacting-domain death agonist (*BID*) were affected by gestation (Fig. 3i, j, k, respectively).

### Mitochondrial complexes, respiration, and content

**Fig. 4:**
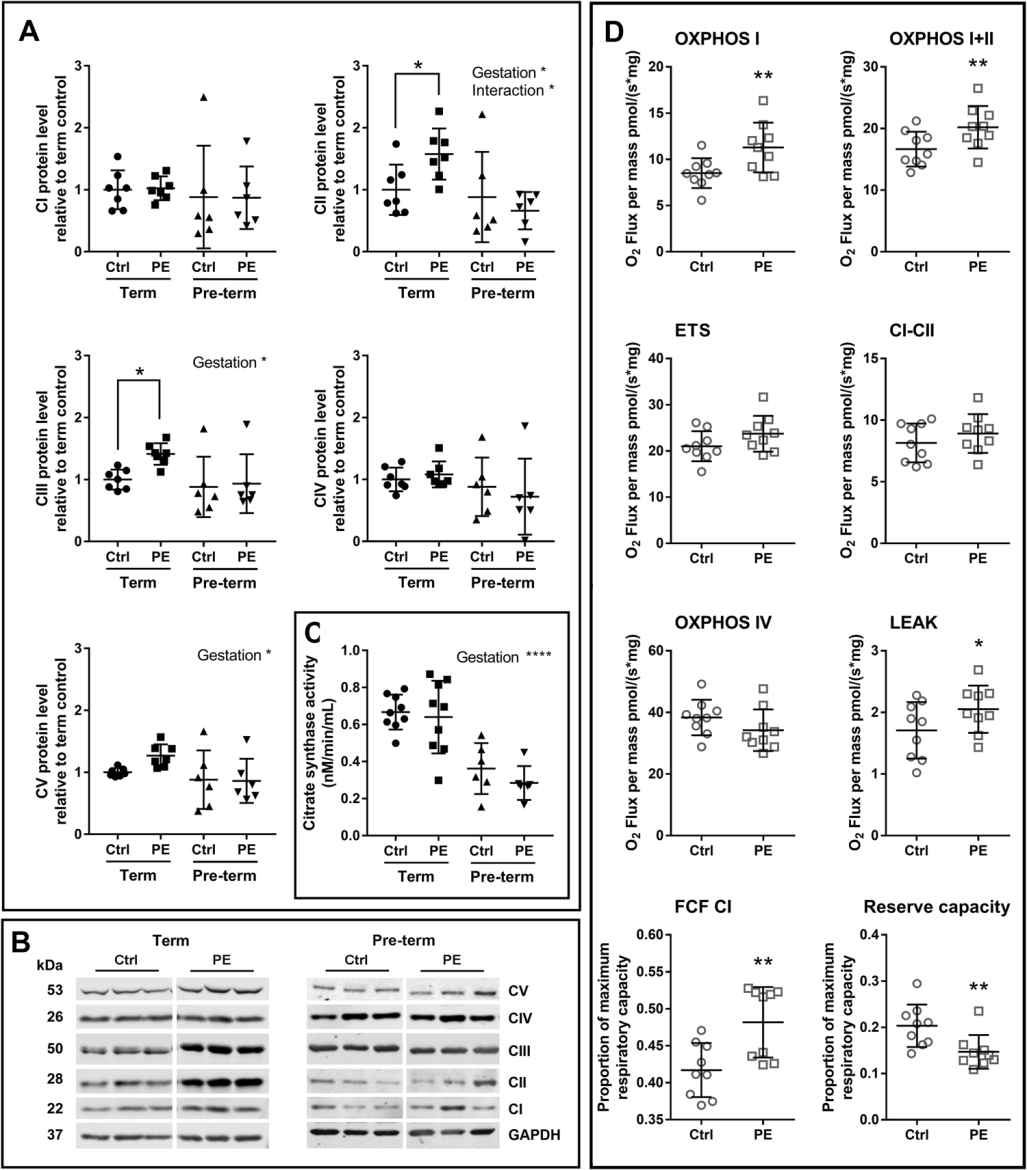
Mitochondrial complex levels and respiration are dysregulated in preeclamptic placentae. **a** Protein levels of mitochondrial complexes II and III were increased in preeclamptic placentae from term pregnancies compared to control placentae from term pregnancies (*p* = 0.0360, 0.0369, fold change 1.576, 1.413, respectively). Complex II was affected by gestation (*p* = 0.0125) and interaction factor (*p* = 0.0478), complex III was affected by gestation (*p* = 0.0401), and complex V (ATP synthase) was affected by gestation (*p* = 0.0370). There was no change in the protein level of complexes I, IV and V in placentae from term pregnancies, or all complexes in placentae from pre-term pregnancies. **b** Representative western blot images of mitochondrial complexes. **c** Citrate synthase activity was affected by gestation (*p* < 0.0001, fold change 0.495 pre-term compared to term pregnancies). **d** Oxidative phosphorylation through mitochondrial complexes I (OXPHOS I) and I + II, and non-phosphorylating LEAK respiration, were increased in term preeclamptic placentae (*p* = 0.0084, *p* = 0.0199, *p* = 0.0310, fold change 1.188, 1.232, 1.150, respectively). Maximum capacity of the respiratory system (ETS), oxygen consumption through complex II (CI-CII) and OXPHOS IV were not different. OXPHOS I as a proportion of maximal respiratory capacity (FCF CI) was increased in term preeclamptic placentae (*p* = 0.0026, fold change 1.126). Reserve capacity was reduced in term preeclamptic placenta (*p* = 0.0042, fold change 0.745). GAPDH = Glyceraldehyde 3-phosphate dehydrogenase. *N* = 6–10; *P < 0.05; **P < 0.01, ***P < 0.001

The relative proportions of mitochondrial complexes (CI–CV) can influence ETS efficacy. Complexes II and III were increased in term preeclamptic placentae, but unchanged in pre-term preeclamptic placentae, compared to controls (Fig. 4a and b). Citrate synthase activity relates to mitochondrial function/content, and was not different between preeclamptic placentae and controls, but affected by gestational age (Fig. 4c). The mtDNA/nDNA ratio estimates mitochondrial content, and was increased in term preeclamptic placentae compared to controls (*p* = 0.0093, fold change 1.458). There was no difference in total placental weight in term preeclamptic placentae compared to controls (control 0.62 ± 0.11 kg, preeclamptic 0.68 ± 0.11 kg).

The production of ATP is achieved through oxidative phosphorylation (OXPHOS) via ETS complexes. To better characterise the effect of changes in relative proportions of mitochondrial complexes in term preeclamptic placentae, mitochondrial respiration was examined in these tissues. There was an overall increase in mitochondrial respiration in term preeclamptic placentae compared to controls (Fig. 4d). Oxidative phosphorylation through mitochondrial complexes I (OXPHOS I) and I + II, and non-phosphorylating LEAK respiration, were increased in term preeclamptic placentae compared to controls. OXPHOS I as a proportion of maximal respiratory capacity was increased in term preeclamptic placentae, and reserve capacity (measure of mitochondrial ability to respond to increased energy demand) was reduced in term preeclamptic placentae compared to controls (Fig. 4d).

### Increased mitochondrial respiration and production of hydrogen peroxide post in vitro hypoxia/reoxygenation

**Fig. 5:**
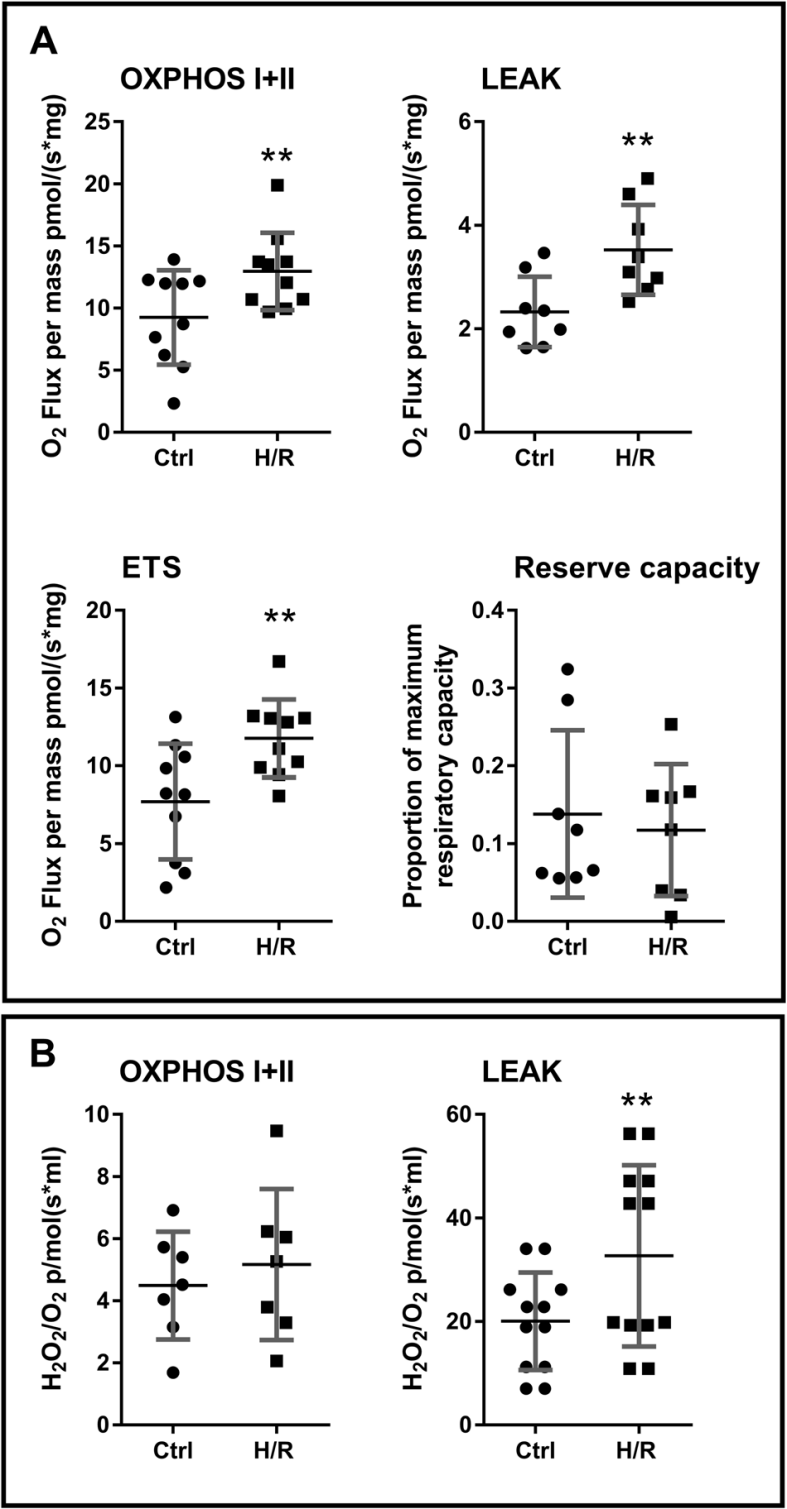
In vitro hypoxia/reoxygenation of placental tissue leads to increased mitochondrial respiration and increased production of hydrogen peroxide during LEAK state respiration. **a** Oxidative phosphorylation through mitochondrial complexes I + II (OXPHOS I + II), non-phosphorylating LEAK respiration, and maximum capacity of the respiratory system (ETS), were increased in healthy placental tissue after hypoxia/reoxygenation (*p* = 0.0065, *p* = 0.0091, *p* = 0.0060, fold change 1.401, 1.516, 1.527, respectively). Reserve capacity was not changed. **b** Hydrogen peroxide production was not changed during OXPHOS I + II, and was increased in LEAK respiration in healthy placental tissue after hypoxia/reoxygenation (*p* = 0.0020, fold change 1.945). *N* = 11; **P < 0.01

To investigate the direct effect of hypoxia/reoxygenation on placental tissue, healthy term placentae were subjected to hypoxia followed by reoxygenation in vitro while measuring mitochondrial respiration and H_2_O_2_ production. Similar to that observed in term preeclamptic placentae (Figs. 1a and 4c), there was an increase in H_2_O_2_ production and mitochondrial respiration post hypoxia/reoxygenation in healthy placentae compared to matched tissue that did not undergo hypoxia/reoxygenation (Fig. 5). OXPHOS I + II, LEAK respiration, and maximum capacity of the respiratory system, were increased in healthy placental tissue after hypoxia/reoxygenation, reserve capacity was not changed (Fig. 5a). Hypoxia/reoxygenation of healthy placental tissue did not change H_2_O_2_ production during OXPHOS I + II, but H_2_O_2_ production was increased during LEAK respiration (Fig. 5b).

## Discussion

Preeclampsia is a dangerous pregnancy disorder with symptoms that range in severity^5^. The broad symptomatic range may represent different aetiologies/progression paths leading to different outcomes^4^. This study aimed to characterise key pathways that may contribute to disease severity by investigating placental adaptations in preeclamptic pregnancies that successfully reached term delivery compared to those that did not. Placentae from term preeclamptic pregnancies displayed multiple adaptations in mitochondrial function and related processes that were only minimally observed in preeclamptic pregnancies that delivered pre-term. In contrast, pre-term preeclamptic placentae showed reduced antioxidant function, and differences in immune and apoptotic signalling. Further, we show that hypoxia/reoxygenation of healthy tissue leads to direct and rapid changes in placental mitochondrial function that are similar to those observed in placentae from term preeclamptic pregnancies. Our findings suggest that the placental adaptations that determine disease severity and allow pregnancies to progress to term involve antioxidant responses and dynamic mitochondrial-related processes.

### An increased antioxidant response is associated with preeclamptic placentae that reach term

Oxidative stress is a feature of disorders related to ischaemia/reperfusion injury^36^. Preeclampsia is thought to be associated with placental ischaemia/reperfusion, and increased markers of oxidative stress have been measured in preeclampsia^6,7,19,37,38^. In the current work, we have found that placentae from term preeclamptic pregnancies had increased levels of the ROS H_2_O_2_, but they also appear to have a compensatory antioxidant response, with increased total antioxidant activity that may be partly driven through increased GPx function. In contrast, placentae from pre-term preeclampsia did not exhibit this antioxidant response, with reduced SOD function, as well as evidence of a greater immune response, with increased NF-κB. This decreased antioxidant function and increased immune response in more severe preeclampsia is in agreement with previously published data^19,39–43^. The decreased mitochondrial SOD activity in pre-term preeclamptic placentae may have led to the lower levels of H_2_O_2_ in these tissues compared to pre-term controls, as SOD converts superoxide to H_2_O_2_. However, a portion of the control pre-term pregnancies experienced labour (compared to none of the pre-term preeclamptic pregnancies; Table 1), and the relative increase in H_2_O_2_ may also be related to this factor. Further, it is possible that the differences in antioxidant function over gestation (decreased total SOD activity, and increased catalase, GPx and total antioxidant activity at term) are part a normal progression to delivery, or are due to the complications of the pre-term pregnancies. Additionally, tissues used in this investigation came from an overweight population (Table 1). Although body mass index was not different between the control and preeclamptic populations in this study, high adiposity is a known risk factor for preeclampsia, and the outcomes observed here may be different in a lean population. In term preeclampsia there was also no change in the ROS-responsive secretase *ADAM10*^44^. *ADAM10* has previously been observed to increase in pre-term preeclampsia, and through its action in protein secretion may contribute to increased placental shedding of soluble fms-like tyrosine kinase 1 (sFlt1); a factor thought to play an important role in preeclampsia pathogenies^45–48^. The lack of change found in *ADAM10* in the current investigation suggests that term preeclamptic pregnancies may not exhibit the same level of protein misfolding observed in pre-term tissue^45^. The ability to counter ROS may therefore represent a successful adaptation of placentae from the pregnancies with preeclampsia that reach term, allowing the placenta to continue to function under adverse conditions. Indeed, in term pregnancies complicated by maternal asthma, placental enzymatic antioxidant capacity is increased^49^, suggesting a compensatory response to oxidative stress occurred in placentae from these otherwise healthy pregnancies.

### Mitochondrial-related adaptations: fission and fusion

ROS signalling and mitochondrial function may be important in the response of preeclamptic placentae that reach term. Mitochondria undergo constant morphological change through cycles of fission and fusion, which are part of the mitochondrial response to environmental conditions, including stress^9,50,51^. These adaptive processes likely contribute to whether a tissue responds to minimise damage, or progresses to a more pathological state. The mitochondrial fission/fusion process was differentially disrupted in term and pre-term preeclamptic placentae, indicating that distinct cellular damage/repair pathways are activated in the different forms of the disorder. A pro-fusion environment was present in term preeclamptic pregnancies, with decreased pro-fission FIS1 and increased pro-fusion L-OPA1 and MFN1, whereas no changes in fission/fusion regulator proteins were observed in pre-term preeclamptic pregnancies. This contrasts other studies, which have reported increases in the pro-fission regulator OPA1 and decreases in the pro-fusion regulator DRP1 in early onset preeclampsia^52,53^. However, other fission/fusion regulators including mitochondrial fission factor^53^, MFN1 and MFN2^52^, were not found to be changed in preeclamptic placentae in these studies, suggesting that the regulation of mitochondrial fission/fusion in the placenta is complex. Fission allows sequestering of damaged mitochondria for disposal by autophagy, whereas fusion can allow recovery of damage by fusing lower functioning mitochondria to better functioning parts of the reticular network^9^. Therefore, mitochondrial fusion may also have contributed to the ongoing successful function of the placenta in preeclamptic pregnancies that reached term.

### Mitochondrial-related adaptations: apoptosis

Preeclampsia has been associated with increased markers of apoptosis in trophoblasts/the placenta^41,54–57^, and we also observed an increase in the pro-apoptotic BAX/BCL2 ration in pre-term preeclamptic placentae. In placenta from term preeclampsia, we found a decrease in pro-apoptotic BAX and an increase in anti-apoptotic BCL2, as well as no change in CASP9, demonstrating active mitochondrial suppression of apoptotic signalling and suggesting that mitochondria promote cell survival in these placentae.

Although we observed evidence of mitochondrial apoptotic signalling suppression, other mechanisms may signal apoptosis. Mitochondrial/ER crosstalk is important in stress mediated apoptosis^58^. We found no preeclampsia-related changes in *BAP31*, *CASP8* or *BID*, which can signal apoptosis through the extrinsic pathway and via the ER. Apoptotic signalling in preeclamptic placentae may be regulated by ER associations^59^, but determining the level of ER involvement in preeclampsia requires additional investigation. Further, the outer layer of the human placenta is a multinuclear syncytium (the syncytiotrophoblast), in which the apoptotic cascade may have specific functions in the progression of cell fate^60^.

### Mitochondrial-related adaptations: respiration and content

Given the evidence of mitochondrial salvage in placentae from term preeclampsia (discussed above), we investigated if these mitochondria functioned normally. The organisation of ETS complexes determines mitochondrial energy efficiency^61,62^. We observed changes in the relative levels of ETS complexes in term preeclamptic placentae, suggesting that there may be effects on mitochondrial efficiency. There was no change in citrate synthase activity between control and preeclamptic placentae, suggesting overall mitochondrial tissue function was not altered in preeclamptic placentae. Of note, citrate synthase activity was reduced in pre-term relative to term tissue, suggesting that placental mitochondrial function changes over this gestational period. Indeed, we have previously shown that mitochondrial respiration changes over early gestation^27^. However, all pre-term tissue is necessarily from complicated pregnancies, and pathology in the pre-term control group may impact on mitochondrial related functions. Future work will be required to investigate if there are changes in mitochondrial respiration and other functions in pre-term tissues. Term preeclamptic tissues had higher mitochondrial respiratory levels; however, there was a decrease in the reserve capacity, indicating mitochondria are routinely functioning closer to their maximum capacity. Changes in ETS complex levels could disrupt the formation of mitochondrial supercomplexes and result in increased production of ROS^63^. Therefore, increased respiration through a dysfunctional ETS may have led to the increased levels of H_2_O_2_ observed in preeclamptic tissue. Mitochondrial respiration can be increased in skeletal muscle in response to exercise as an adaptation to greater energy demands^64^, and the increase in respiration observed in preeclamptic placentae in the current study may represent a mechanism by which the tissue is able to maintain overall function with sub-optimal mitochondrial function.

### Mitochondrial respiration and reactive oxygen species production increase after hypoxia/reoxygenation

The pathophysiology of preeclampsia is thought to be associated with aberrant maternal blood flow to the placenta^6,7^. Disrupted oxygen levels have been proposed to lead to placental dysfunction^6^, but may also be a trigger for the adaptations observed in the current study. In vitro exposure of healthy placental tissue to hypoxia and reoxygenation leads to a rapid increase in respiration, demonstrating that placental mitochondria are capable of responding to variations in oxygen supply even over short time periods. Indeed, we have previously shown that labour is associated with increases in placental mitochondrial respiration^27^. Others have observed increased markers of oxidative stress in laboured tissue^65–68^, suggesting a direct link between placental mitochondrial respiration and oxidative damage. In the current investigation, we also found an increase in H_2_O_2_ production with hypoxia/reoxygenation, demonstrating that hypoxia/reoxygenation can be a direct source of ROS, which may be both damaging or act in signalling.

## Conclusions

**Fig. 6:**
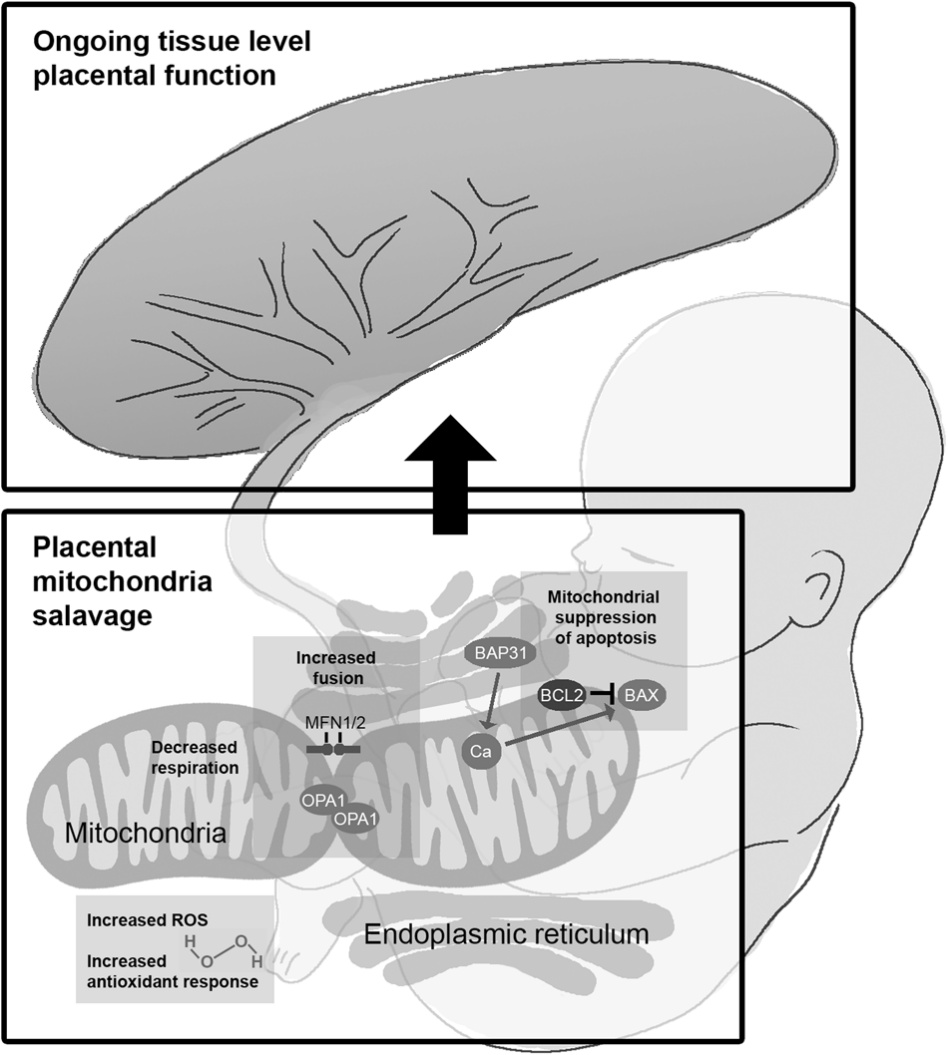
Summary diagram showing proposed mechanisms of mitochondrial-related adaptations in preeclamptic placentae that reach term delivery. Increased reactive oxygen species (ROS) production leads to a compensatory increase in antioxidant levels, potentially protecting mitochondria and other cellular organelles. Mitochondria show evidence of damage, with lower mitochondrial respiratory efficacy and changes in the relative proportions of mitochondrial electron transfer system complexes. To compensate for this damage, mitochondrial dynamics is shifted to increased fusion, allowing the salvage of damaged mitochondria, and there is mitochondrial suppression of apoptotic signalling. Although mitochondria show evidence of dysfunction, salvaged mitochondria contribute to ongoing tissue function, allowing for pregnancy continuation

We show multiple mitochondrial-related adaptions in placentae from preeclamptic pregnancies that reached term, which are not found in preeclamptic pregnancies that delivered pre-term (Fig. 6). ROS signalling in term preeclamptic placentae may be at a level to trigger compensatory antioxidant and mitochondrial responses, allowing tissue level maintenance of function when there is organelle level dysfunction. In other tissues, mitochondrial ROS signalling is known to lead to a range of physiological responses (including increased antioxidant defences, activation of potassium channels, activation of uncoupling proteins, and expression of pro-survival genes) that can precondition improved cell survival and limit ischaemia/reperfusion injury^36^; determining if these systems are also active in the placenta requires further investigation. Additionally, the human placenta has distinct functional cell types—cytotrophoblasts and the syncytiotrophoblast—that possess mitochondria with very different structures and functions^20^. Further examination is required into the regulation of mitochondrial-related processes such as fission/fusion and apoptosis in cytotrophoblasts and the syncytiotrophoblast, and how these processes may differ in these important cell types.

## Electronic supplementary material

Supplementary figure 1

Supplementary figure 2

Supplementary figure 3
