# Supplementary figures and images for: Placental mitochondrial adaptations in preeclampsia associated with progression to term delivery
Source: Cell Death Dis. 2018 Nov 19;9(12):1150. doi: 10.1038/s41419-018-1190-9 (PMC6242930; doi:10.1038/s41419-018-1190-9)

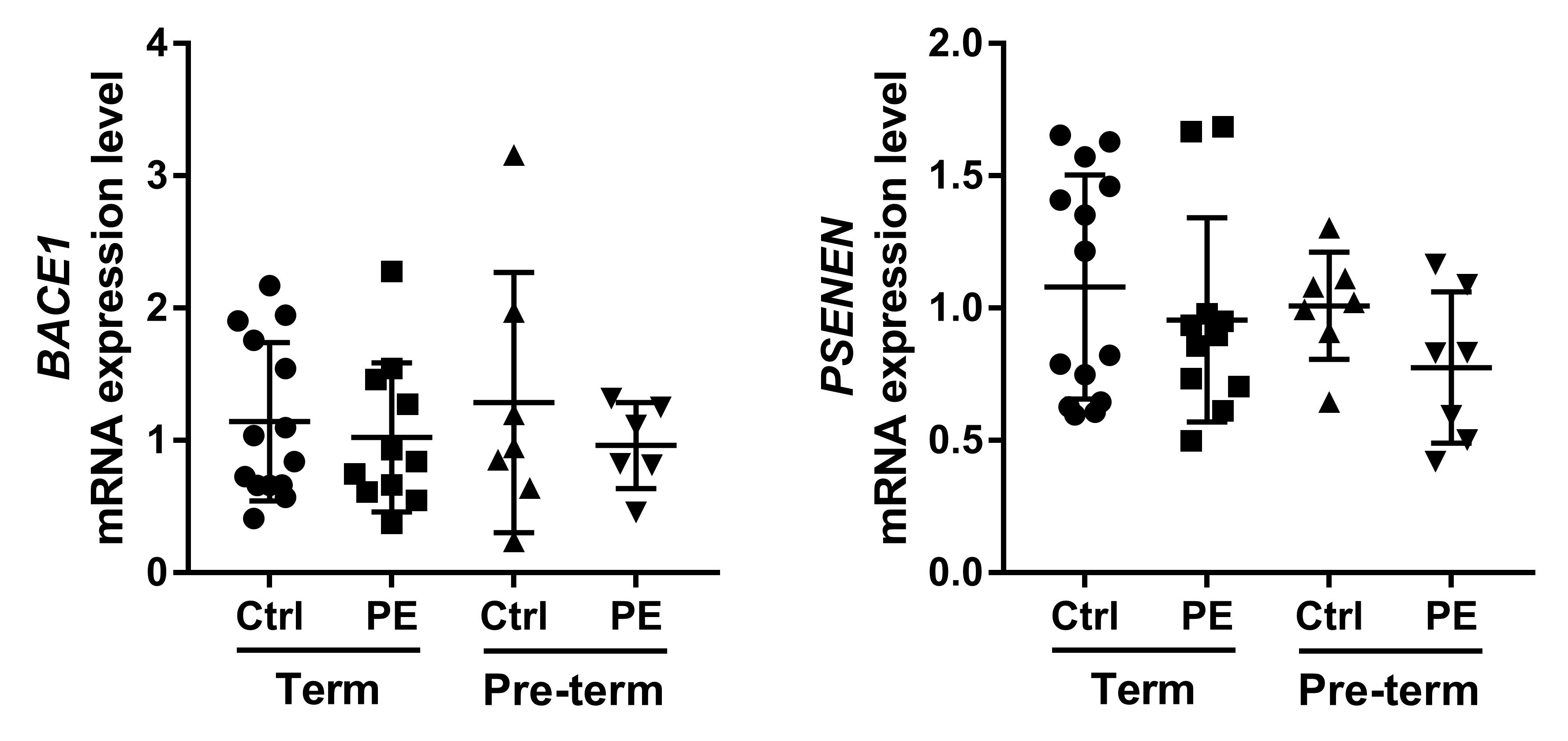

Supplement: Supplementary file 1 — Supplementary figure 1 [file 41419_2018_1190_MOESM1_ESM.tif]

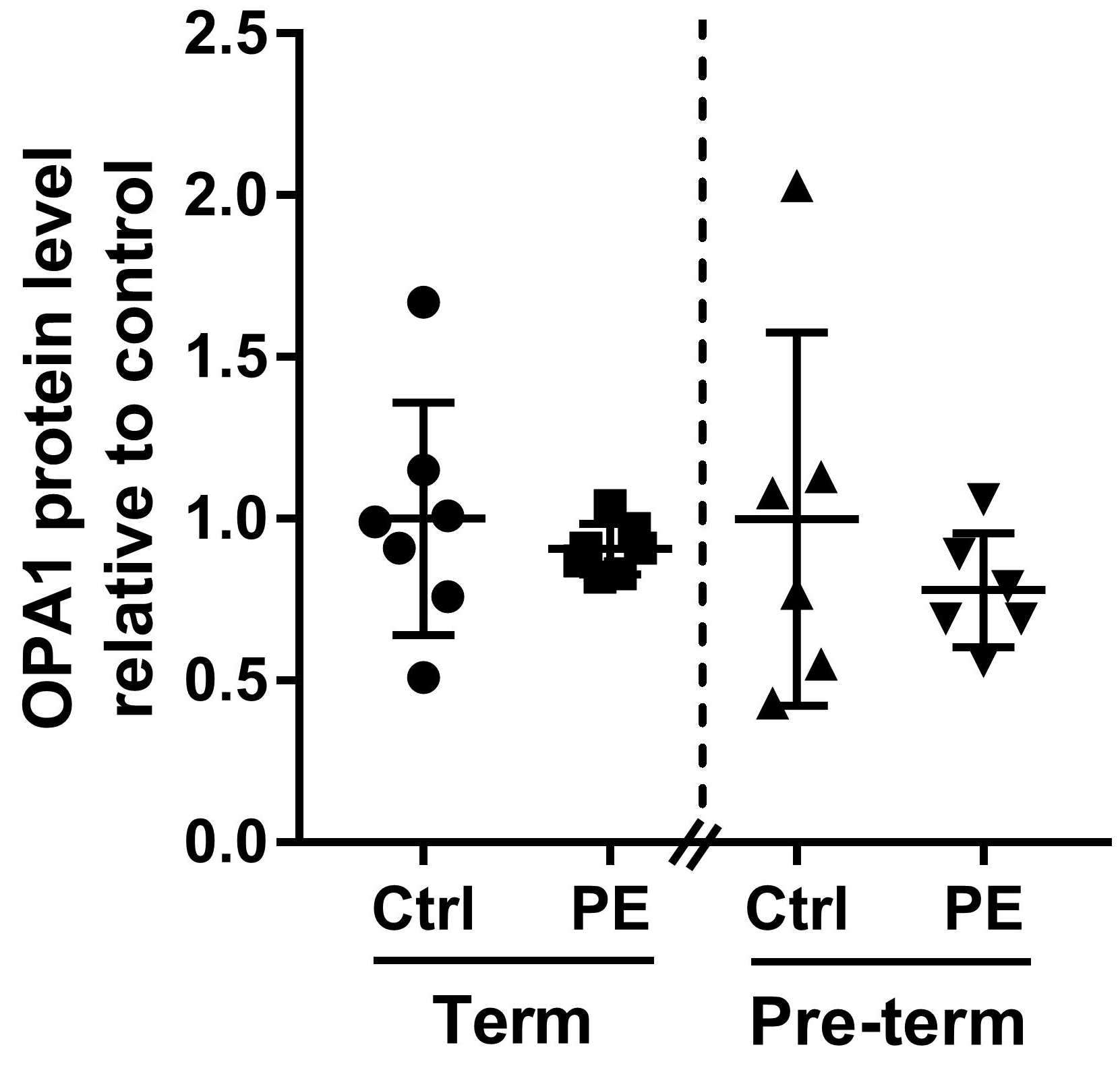

Supplement: Supplementary file 2 — Supplementary figure 2 [file 41419_2018_1190_MOESM2_ESM.tif]

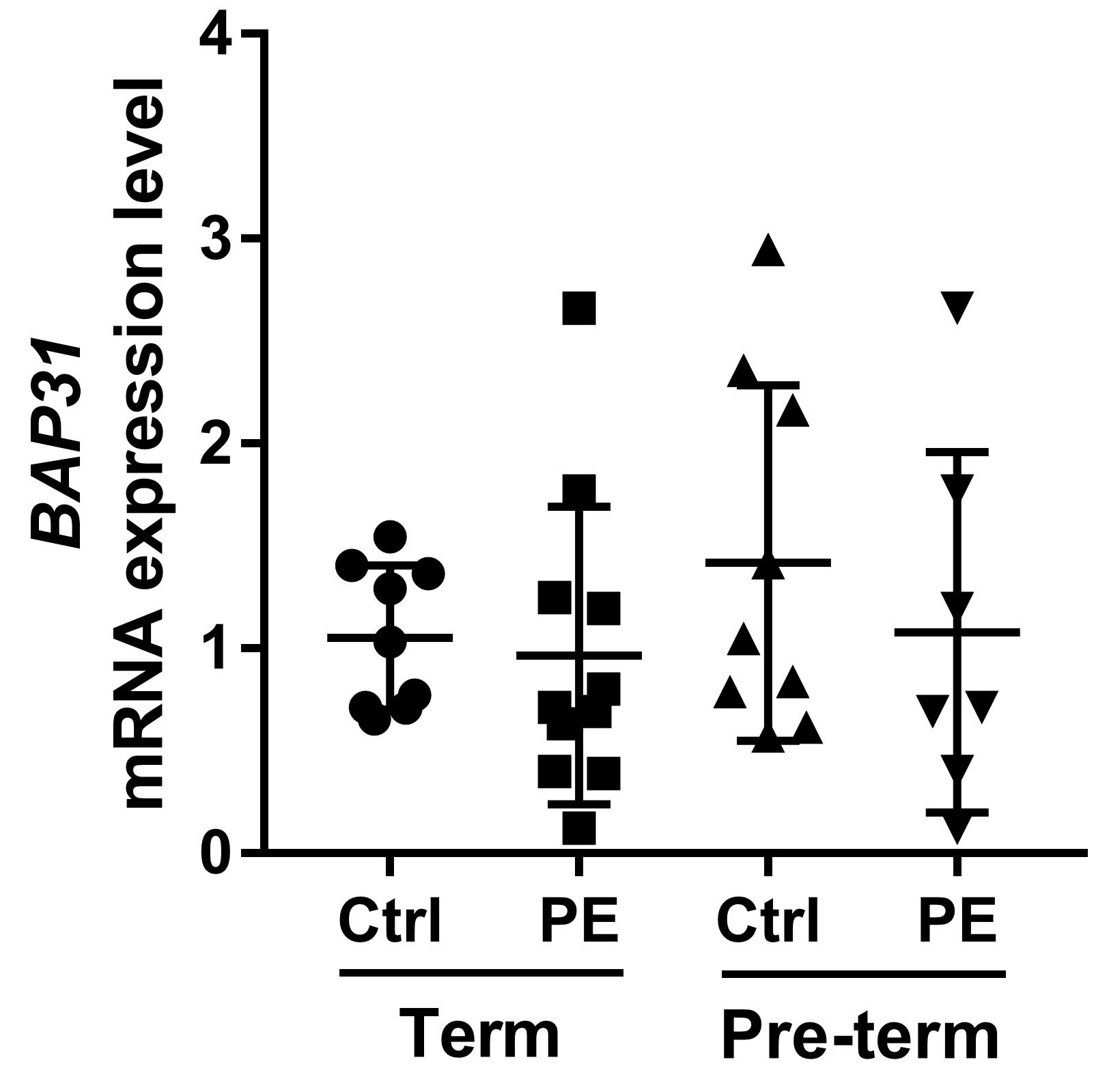

Supplement: Supplementary file 3 — Supplementary figure 3 [file 41419_2018_1190_MOESM3_ESM.tif]
